# Evaluating vacquinol-1 in rats carrying glioblastoma models RG2 and NS1

**DOI:** 10.18632/oncotarget.23842

**Published:** 2018-01-03

**Authors:** Jonatan Ahlstedt, Karolina Förnvik, Shaian Zolfaghari, Dongoh Kwak, Lars G.J. Hammarström, Patrik Ernfors, Leif G. Salford, Henrietta Nittby Redebrandt

**Affiliations:** ^1^ Rausing Laboratory, Division of Neurosurgery, Department of Clinical Sciences Lund, Lund University, Lund, Sweden, Lund University, Sweden; ^2^ Department of Medical Biochemistry and Biophysics, Division of Molecular Neurobiology, Karolinska Institutet, Stockholm, Sweden; ^3^ Chemical Biology Consortium Sweden, Science for Life Laboratory, Department of Medical Biochemistry and Biophysics, Division of Molecular Translational Medicine and Chemical Biology, Karolinska Institutet, Stockholm, Sweden

**Keywords:** glioblastoma, vacquinol-1, RG2, NS1, rat

## Abstract

Glioblastoma multiforme (GBM) is the most common malignant primary brain tumor, and available experimental and routine therapies result in limited survival benefits. A vulnerability of GBM cells to catastrophic vacuolization and cell death, a process termed methuosis, induced by Vacquinol-1 (VQ-1) has been described earlier. In the present study, we investigate the efficacy of VQ-1 treatment in two syngeneic rat GBM models, RG2 and NS1. VQ-1 treatment affected growth of both RG2 and NS1 cells *in vitro*. Intracranially, significant reduction in RG2 tumor size was observed, although no effect was seen on overall survival. No survival advantage or effect on tumor size was seen in animals carrying the NS1 models compared to untreated controls. Furthermore, immunological staining of FOXP3, CD4 and CD8 showed no marked difference in immune cell infiltrate in tumor environment following treatment. Taken together, a survival advantage of VQ-1 treatment alone could not be demonstrated here, even though some effect upon tumor size was seen. Staining for immune cell markers did not indicate that VQ-1 either reduced or increased host anti-tumor immune response.

## INTRODUCTION

Glioblastoma multiforme (GBM) or grade IV astrocytoma is the most common primary brain tumor with a prevalence of approximately 3 cases per 100 000 [1]. It is also one of the most aggressive among brain tumors. With the standard treatment consisting of surgical resection of the tumor if possible, radiotherapy and chemotherapy with concomitant and adjuvant Temozolomide (TMZ), the average survival time is approximately 15 months [2]. Diverse factors such as age, Karnofsky performance score, tumor localization and mutational pattern of the O6-alkylguanine DNA alkyltransferase (MGMT) promoter affect the prognosis. One approach in glioblastoma research has been to develop targeted therapies. However, GBM are highly diversified tumors, with both gain- and loss-of-function in core signaling pathways, which is a considerable obstacle to this approach. For example, preclinical data suggests targeting the epidermal growth factor receptor (EGFR) or vascular endothelial growth factor receptor (VEGFR) to be a promising approach, but has not lead to any breakthrough in clinical trials [3, 4]. Therefore, it would be attractive to find a more general function gained by GBM cells that is present in most tumor cells and patients.

Oncogenic mutations elevating membrane activity and vacuolization drive anabolic metabolism creating dependency on nutrient influx through macropinocytosis [5]. Nutrient scavenging to support proliferation and survival of cancer cells was recently included as one of the hallmarks of cancer metabolism [6]. Mutations in *ras* genes occur in approximately 30% of human malignancies and genomic profiling of GBM has shown that *ras* is mutated in nearly all tumors [7]. Notably an introduction of the mutation H-Ras (G12V) triggers macropinocytosis, accumulation of cytoplasmic vacuoles and cell death without caspase activation or DNA fragmentation in human glioblastoma, gastric carcinoma, pancreatic carcinoma and neuroblastoma cells [8, 9]. These vacuoles are phase-lucent and lysosome-associated membrane protein 1 (LAMP1)-positive, clearly distinct from lysosomes, autolysosomes, and late endosomes, which typically contain electron-dense organelle remnants or degraded cytoplasmic components. Furthermore, the vacuoles are of varying size, mostly empty and bounded by a single membrane, unlike clathrin-coated endosomes which are regular in size and are bounded by a double membrane [7, 8]. Hence, taking advantage of the elevated nutrient scavenging by inducing vacuolization and/or interfering with vacuole clearance may be predicted to result in a relatively selective cytotoxicity of cancer cells. This is proposed as a non-apoptotic mechanism of cell death and has been termed methuosis [10].

Several studies have identified lipophilic compounds that induce cellular vacuolization of cancer cells, resulting in metabolic catastrophe and cell death [8, 11]. One such compound, Vacquinol-1 (VQ-1), was identified by screening two human glioma cell lines for changes after exposure to a large set of compounds (NIH diversity set II). Animal trials on xenografted human gliomas in mice showed promise for extending overall survival but were not reproducible [P. Ernfors, pers. comm.]. *In vitro*, VQ-1 exposure led to accumulation of large vacuoles, ATP-depletion and eventually results in methuosis-like membrane rupture and cell death that is not seen in fibroblast cells or other cell types such as breast, prostate, bladder, and neuroblastoma cell lines. There is ongoing research detailing the oncolytic effect and brain exposure of different isomers of VQ-1 [12], and the effect of VQ-1 is suspected to be counter regulated by exogenous ATP, activating the transient receptor potential cation channel, subfamily M, member 7 (TRPM7) [13].

GBM cells create an immunosuppressive microenvironment and employ various methods to escape immune surveillance, and immunomodulating therapy is an extensive area of research [14]. Therefore, we wished to examine the effect of VQ-1 treatment in a fully immunocompetent tumor model. Since rats have a different, and often reduced, capacity to tolerate potentially highly effective but also toxic drugs as compared to mice, we also wanted to test different treatment protocols.

In the present study, two different rat GBM models were used to test the effect of orally administered VQ-1. Overall survival and tumor size were studied. Additionally, representative material was investigated for markers of immune response or suppression, including CD4, CD8 and FOXP3, to attempt to visualize infiltration of immune cells into the tumor environment.

## RESULTS

### Cytotoxicity of VQ-1, in RG2 and NS1 cells

**Figure 1:**
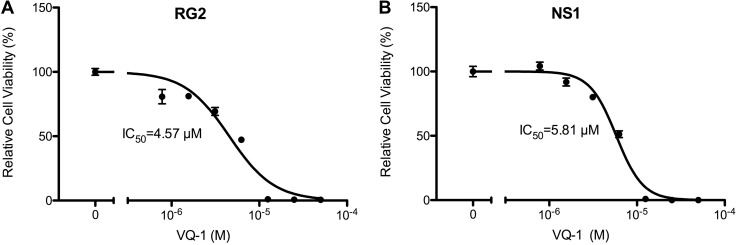
Graph of relative cell viability with vacquinol-1 (**A**) Relative cell viability of RG2 cells *in vitro* with VQ-1. (**B**) Relative cell viability of NS1 cells *in vitro* with VQ-1.

**Figure 2:**
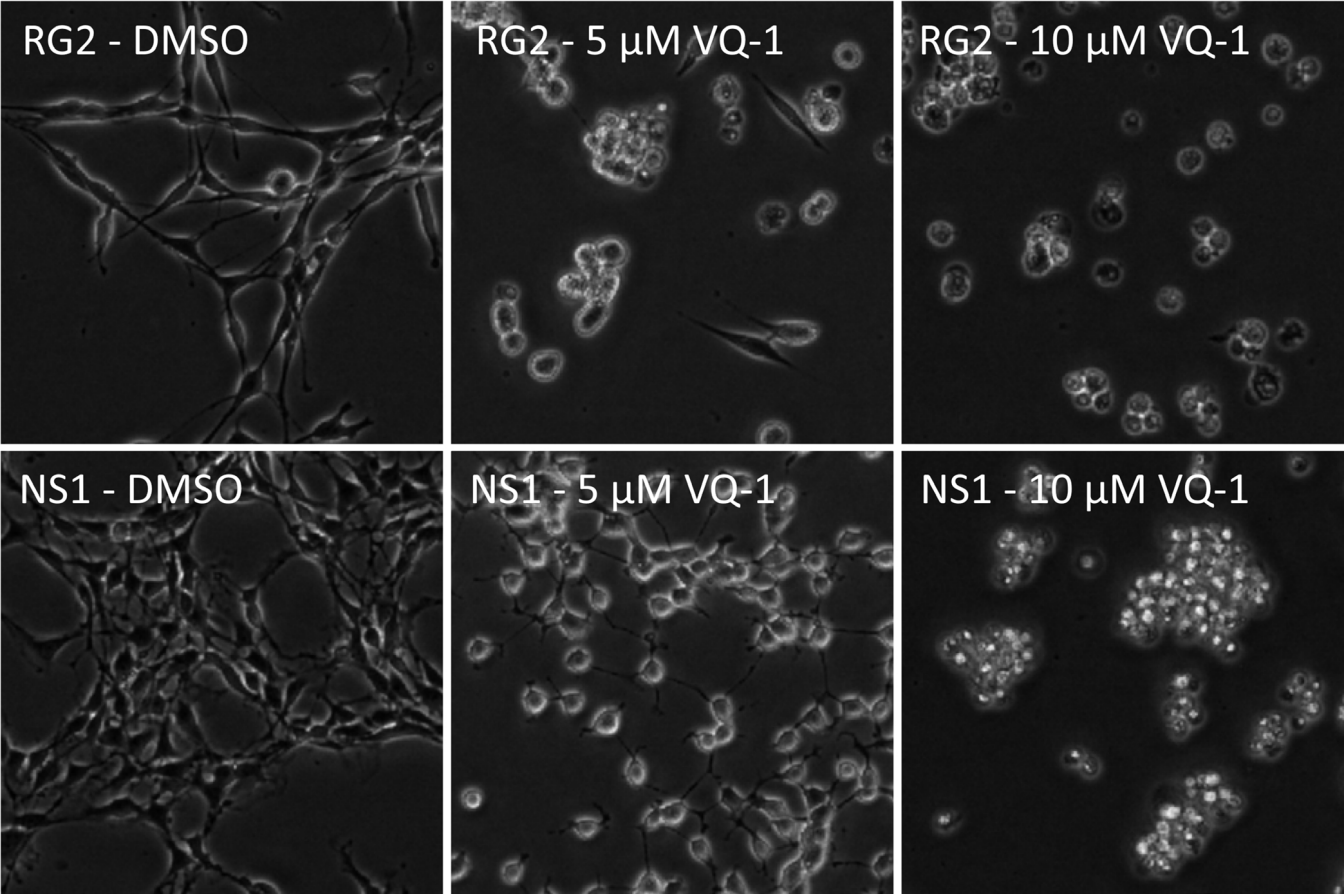
Microscopic images showing RG2 (top row) and NS1 cells (bottom row) exposed to increasing concentrations of VQ-1, 5 µM (center column) and 10 µM (right column) respectively

*In vitro* dose-response measurements of RG2 and NS1 cells showed an IC_50_ of 4.57 µM and IC_50_ of 5.81 µM, respectively (Figure 1). Microscopic images of RG2 and NS1 exposed to 5 µM and 10 µM VQ-1 concentration are displayed in Figure 2. This is within the range of that observed for human glioma cells U3013. VQ-1 pharmacokinetics has shown adequate bioavailability and good penetrance of the blood brain barrier *in vivo* after intravenous and oral administration. When testing *in vivo* peroral administration in mice, a maximal plasma exposure of 3,279 ng/ml was obtained and an exposure of 1,860 ng/ml in the brain with a single dose of 20 mg/kg VQ-1. VQ-1 has shown adequate stability *in vivo* with a t_1/2_ of 52 hours in plasma. We therefore chose a peroral administration route.

### Reduced tumor size but no difference in survival in rats carrying RG2 tumors

Syngeneic animals were inoculated with 5 000 RG2 cells in the right caudate nucleus. 18 rats received intracranial inoculations of 5000 RG2 cells and were divided into two groups (VQ-1 *n* = 9, controls *n* = 9). Based on previously observed progression rate of the RG2 model [14], cutoff was set at 28 days, at which time the remaining rats were killed for histological examination. Five animals from the control group and four animals from the treated group were euthanized at the time of cutoff.

**Figure 3:**
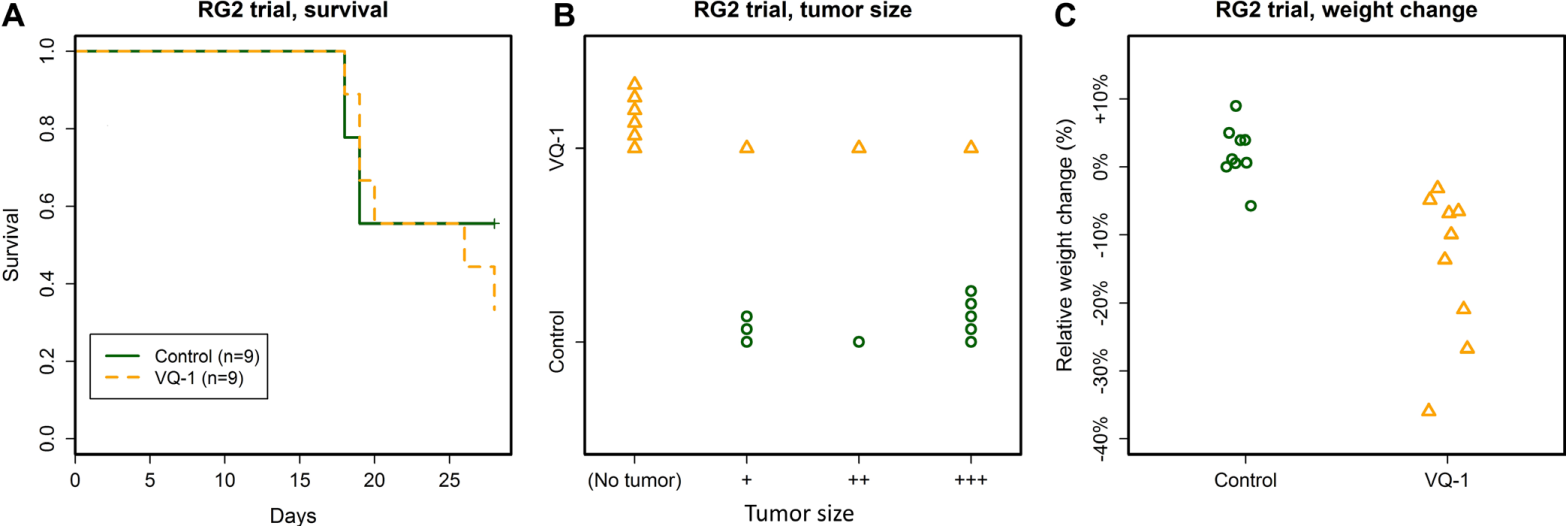
Summary of the RG2 trial (**A**) No effect of VQ-1 treatment was seen on survival in rats with RG2 tumors at the 28-day cutoff. Mean survival time in control animals was 23.8 ± 5 days and in VQ-1 treated animals 23.8 ± 4.6 days. (**B**) Tumors were significantly smaller in the VQ-treated group as compared to controls (*p* = 0.006). For examples of the tumor size grading, please refer to supplementary materials. (**C**) Body weight was significantly decreased in the VQ-treated group compared to controls (*p* = 0.001).

Survival curves between the treatment and control groups showed no difference (Figure 3A). In animals receiving VQ-1, significant loss of body weight occurred (relative weight change 0.14 ± 0.11, abs. weight change from 168 ± 9 g to 144 ± 21 g), compared to controls, in which body weight was maintained or increased (relative weight change 0.02 ± 0.04, abs. weight change from 170 ± 16 g to 173 ± 11 g, Figure 3C). In addition to weight loss, several animals in the treated group developed periodical labored breathing, which was not present in any of the animals receiving gavage of vehicle only. The animals displayed otherwise normal behavior.

At the cutoff time of the trial at 28 days post tumor graft, four treated rats and five control rats were alive. None of the surviving treated animals had visible tumors upon examination, while all control animals carried visible tumors. Across the entire trial, the VQ-1 treated animals had significantly lower tumor size grading as compared to controls (*p* = 0.006, Figure 3B).

### NS1 trials

In a first trial, tumors were established by inoculation of 50 000 NS1 cells in 8 animals. These were divided into two groups, one which received vehicle administration (*n* = 4) and one where VQ-1 was administered at a dose of 50 mg/kg at days 5, 3, 1, 1, 2, 4, 7, 10, and so on every third day, with inoculation performed at day 0 (*n* = 4). All animals were euthanized either day 16 or 17 after tumor cell inoculation due to development of symptoms.

**Figure 4:**
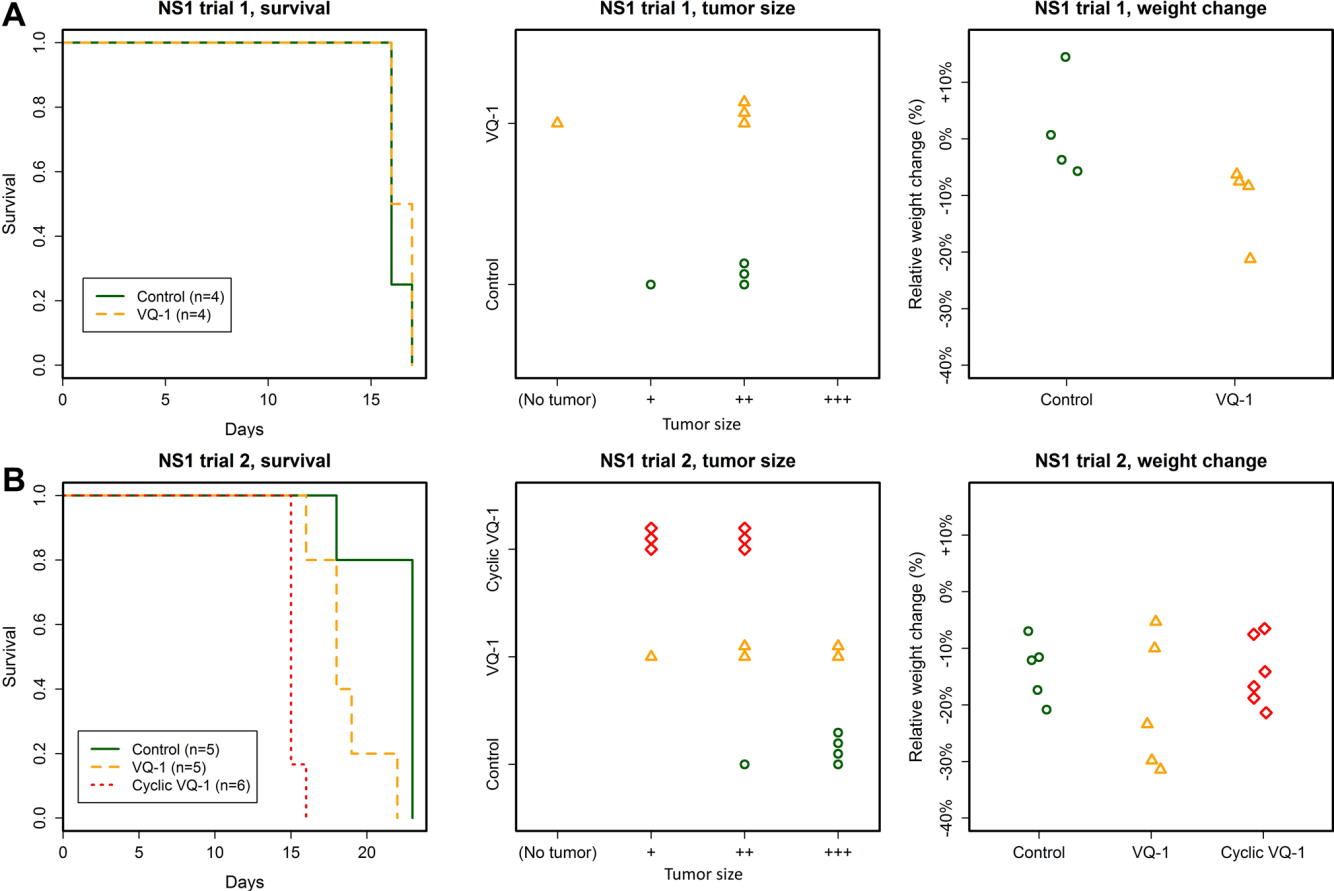
Summary of the NS1 trials (**A**) No effect of VQ-1 treatment was seen in animals with NS1 tumors, regarding survival or tumor size (NS1 inoculation with 50 000 cells). Mean survival in VQ-treated rats was 16.4 ± 0.5 days and in control animals 16.2 ± 0.5 days. There was no statistically significant weight difference between the two groups. (**B**) No effect of VQ-1 treatment was seen in animals with NS1 tumors, regarding survival or tumor size (NS1 inoculation with 5 000 cells). Mean survival in VQ-1 treated rats was 18.6 ± 2.2 days for standard protocol and 15.2 ± 0.4 days in the cyclic protocol; as compared to 22 ± 2.2 days for control animals. Since the cyclically treated animals died significantly earlier than control animals, meaningful comparison of tumor size could not be made.

There was no difference in survival between the two groups, and no difference was seen in tumor size upon inspection (Figure 4). With anti-GFP immunohistochemistry we observed the same pattern of infiltrative tumor growth in both treated and untreated animals. Weight loss was observed in the treated group, as in earlier trials (weight change from 142 ± 6 g to 126 ± 9 g in treated animals at time of euthanasia).

In a second trial, a group of animals were inoculated with 5000 NS1 cells. Animals were divided into a control group (*n* = 5), cyclical VQ-1 treatment (*n* = 6) and the same standard regimen as used in the RG2 experiments (*n* = 5), with 70 mg/kg day 7, 8, 10, 13 16 and so on. In the group with cyclical VQ-1 treatment, VQ-1 was perorally administered at 70 mg/kg for five consecutive days preceding a 14-day washout period, after which the 5-day treatment was repeated, with treatment starting 7 days after tumor cell inoculation. All animals were euthanized for ethical reasons due to symptoms of tumor growth or declined general condition.

The animals treated with the cyclical protocol all displayed impaired general condition at days 15–16 after inoculation, approximately 8 days after start of treatment (mean survival 15 days) (Figure 4). Tumor sizes were smaller in this group compared to controls, but these animals died significantly earlier than controls due to side effects of the treatment, making a meaningful comparison of tumor size difficult. Animals in the control group suffered loss of body mass (relative change 0.14 ± 0.05, abs. change from 169 ± 8 g to 146 ± 14), to approximately the same extent as the treated groups (cyclic treatment relative change 0.14 ± 0.06, abs. change 170 ± 3 g to 146 ± 11, standard treatment relative change 0.2 ± 0.12, abs. change 169 ± 6 g to 135 ± 17 g).

### Histology and immune cell infiltration

**Figure 5:**
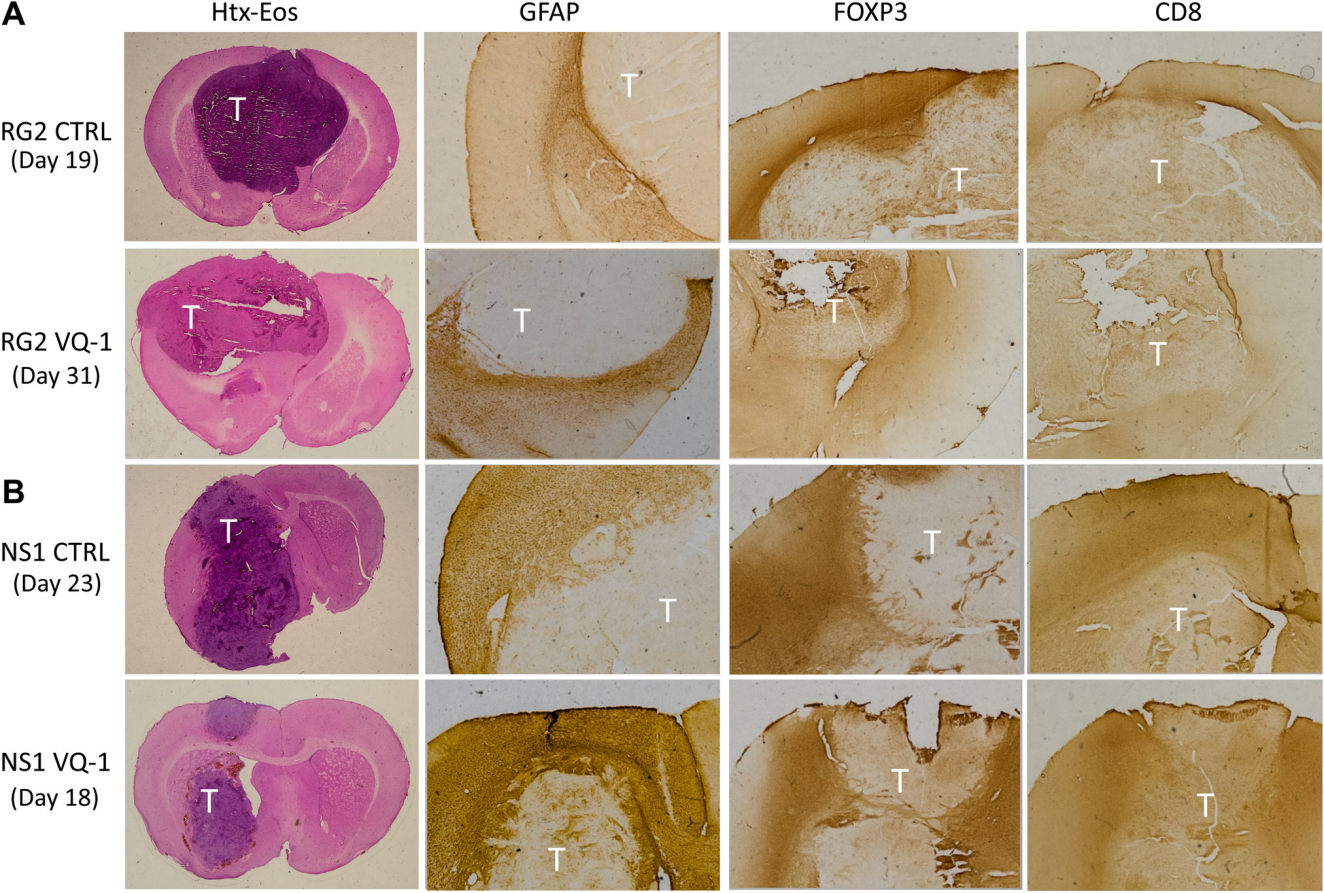
Immunohistochemical staining of excised brains Tumor areas are marked with “T” in images. (**A**) Immunohistochemistry comparing VQ-1 treated RG2 tumors to control with only vehicle. Text to the left indicates at which time the animal was euthanized. No clear difference was noted with any of the performed stainings, but it seems like infiltration of FOXP3 expressing cells is greater in the control situation as compared to the VQ-1 treated animals. (**B**) FOXP3 is present around the tumor in both the control animal and the VQ-1 treated animal. Moreover, GFAP was demonstrated around the tumor front in both situations, and the core seemed to consist of a more necrotic tumor bulk.

Representative animals from treated and untreated groups with both RG2 and NS1 tumors were chosen for immunohistochemical staining for CD4, CD8 and FOXP3 as a way of visualizing immune cell infiltration (Figure 5). The staining showed no apparent difference in intensity or distribution in treated animals compared to controls, in either tumor model used in the trials described, upon visual assessment. Interestingly, when comparing the two tumor models the infiltration of FOXP3-positive cells into the tumor mass seems more prominent in the RG2 tumors compared to NS1. GFAP is present to a larger degree in NS1 tumors compared to RG2, where it seems absent. There were no apparent differences in size or pattern of tumor necrosis in the inspected material, as well as in intensity of staining in these areas. This data suggests VQ-1 lacks any strong immunological or immunomodulatory effect relevant to the current study.

## DISCUSSION

Both RG2 and NS1 are aggressive glioblastoma models used in fully immunocompetent rats, where animals quickly develop symptoms and the number of days available for treatment are reduced as compared to many other models. We could demonstrate an effect of VQ-1 treatment on both NS1 and RG2 glioblastoma cells *in vitro*. However, this study did not find increased survival in rats carrying GBM models treated with VQ-1 *in vivo*. A reduction in tumor size was however observed in the trial using the RG2 model.

VQ-1 is believed to cause necrotic-like cell death of glioblastoma cells and because of this, could be speculated to potentiate an immune response. However, this seems not to be the case in our study. Immunohistochemically staining for GFAP, CD4, CD8, and FOXP3 did not indicate any overt difference between treated and untreated animals. Although it is difficult to compare orthotopic models using human glioblastoma cells in immune-compromised mice with the present more aggressive syngeneic model, results in the present study suggest that immune interactions are at least not greatly facilitated by VQ-1. It is important to bear in mind the methods at our disposal in this paper are limited. Due to the large section thickness an exact quantification of the infiltrating cells is difficult to achieve, and because of this, a visual assessment was done. Immunological evasion plays a big part in tumor development, and activation of the immune system as a means of attacking glioblastomas is an area of much ongoing research. Besides T lymphocytes, which are associated with tumor elimination, brain tumors are characterized by immune infiltrate of dendritic cells, macrophages, microglia and natural killer cells [15–17].

General systemic toxicity of the tested compound hindered dose elevation. Excessive dosing leads to rapid degradation of general condition, weight loss, and respiratory symptoms prompting euthanasia, potentially masking therapeutic effect. This is hinted at by the tumor size difference in trials where survival was the same between groups. Even in the most commonly employed dosage of 70 mg VQ-1 per kg bodyweight in this study induced significant weight loss, and sporadic respiratory issues in treated animals.

With this in mind, in spite of its adequate blood-brain-barrier penetration, an interesting alternative approach would be to explore intratumoral delivery of the VQ-1 compound, potentially reducing or avoiding adverse systemic effects of the treatment. Intratumoral treatment would require either a reservoir implanted into the tumor center, a method that has been described elsewhere [18], or repeated stereotactic injections of the drug through the burr hole used for tumor cell implantation [19]. If proven effective in animal models, intratumoral treatment in the future could possibly be administered to patients who have undergone surgery and where a resection cavity is left.

The VQ-1 used in the present study is a mixture of four stereoisomers. Recently it has been shown that the erythro-isomer (R)-[2-(4chlorophenyl)quinolin-4-yl](2S)-piperidin-2-ylmethanol ([R,S] 2) is superior to the other isomers in terms of efficacy and brain tissue exposure [12]. Treatment with a selected VQ-1 isomer might reduce the amount of VQ-1 needed to see treatment response and in this way also the side effects.

Another possible strategy for further development of VQ-1 could be combination therapies. For example, the efficacy of front-line temozolomide (TMZ) treatment is potentiated by a murine double minute 2 (MDM2) protein-protein interaction inhibitor. MDM2 binds and to and inactivates Nbs1 in the MRE11/Rad50/NBS1 complex and the inhibitor thereby impair DNA repair resulting in potentiation of cell death when administered together with TMZ [20]. Thus, a multipronged approach that targets parallel signaling pathways may converge and act synergistically by increasing cellular susceptibility and compound potency and thereby decreasing effective doses. The effect of VQ-1 may be inhibited by the transient receptor potential cation channel, subfamily M, member 7 (TRPM7) being activated by ATP in the tumor environment [13] and it can be hypothesized that the models used in this study may be vulnerable to a simultaneous administration of TRPM7 inhibitory compounds and VQ-1.

## MATERIALS AND METHODS

### Ethics statement

This study and the procedures described were approved by the animal ethics committee in Lund, with permit ID M44-15 (Nittby Redebrandt). All efforts were made to reduce animal suffering.

### Animals

A total of 42 Fischer 344 rats were included in this study. Rats were purchased from Fischer Scientific. Endpoints were defined as symptoms of brain tumors such as paresis, epilepsy, or poor general condition. Rats were monitored daily with respect to these symptoms, all of which prompted euthanasia. Rats were housed in hutches with enriched environments in groups of two or three with *ad libitum* access to food and water.

### Cell line culture and cytotoxicity measurements

RG2 and NS1 cells were used in this study. RG2 was produced in Fischer 344 rats through ethyl-nitroso-urea (ENU) treatment of pregnant females [21]. It has been considered to be a good experimental model for GBM [22]. RG2 is a particularly aggressive model with short survival from tumor cell inoculation to presentation of symptoms due to tumor growth (19.4 ± 3.8 days) [23]. RG2 is non-immunogenic in syngeneic Fischer rats [22].

NS1 is a new GFP positive tumor cell line that was created by ENU treatment of pregnant homozygous GFP-positive Fischer 344 rats, where the offspring developed GFP-positive CNS-tumors, resulting in the NS1 cell line. Rats inoculated with NS1 cells develop cell-rich tumors with an invasive growth pattern, and since the tumors are GFP-positive, the infiltrative pattern can be studied. The tumors are positive for GFAP, GFP and the tumor cells have been shown to have a strong RNA expression for wt *IDH1*, wt *p53*, *IDO1* and *EGFR* [24].

GBM cells were cultured in RPMI-1640 medium supplemented with 5 ml Na-pyruvate (100 mM stock solution) and 5 ml Hepes (1 M stock solution). Gentamycin (0.5 ml of 50 mg/ml) was added to the nutrient medium to avoid infection.

Cell viability was assessed using CellTiter-Glo (Promega G7571) 48 h post treatment with VQ-1. Luminescence was measured on a FLUOstar Omega microplate reader (BMG Labtech). Viability was calculated as the percentage of control (DMSO treated cells) with at least three replicates for each concentration. IC_50_ was determined as the concentration corresponding half-maximal growth inhibition.

### Inoculation procedure

To establish intracranial tumors, the required number of cells were suspended in 5 µl of R0 medium. Inoculations were performed using a 10 µl Hamilton syringe mounted on a stereotactic frame. Cell suspension was injected through a burr hole placed 2 mm lateral and 1 mm anterior to the bregma, at a depth of 5 mm. The suspension was injected at a pace of 1 µl/min, with a 5-minute pause before retraction at a pace of 1 mm/min. Anesthesia was achieved with continuous isoflurane inhalation. The burr hole was sealed with bone wax and the wound sealed with resorbable suture.

### Vacquinol-1 treatment

VQ-1 was suspended in a vehicle consisting of sodium carbonate (anhydrous), sodium hydrogen carbonate, hypromellose (HPMC), polysorbate 80 and purified water. The compound was administered at a concentration of 10,5 mg/ml for rats treated with the dosage of 70 mg/kg/treatment. In rats treated with the dosage of 30 mg/kg/treatment, a concentration of 4,5 mg/kg was used. Controls received only vehicle, in a volume corresponding to the amount of solution a rat receiving 70 mg/kg at 10,5 mg/ml would have received. The solution was administered by peroral gavage feeding under a light isoflurane sedation. The VQ-1 used in this study was synthesized by Recipharm Ontarget Chemistry, Uppsala, Sweden.

### Protocol for RG2 tumors

Rats receiving VQ-1 treatment had 70 mg/kg administered every third day starting at day 7 after tumor cell inoculation, with a startup period of two consecutive days of treatment, e.g. treatment at days 7, 8, 10, 13, 16, etc. until animals displayed symptoms. Control groups received vehicle only on the same schedule. The experiment was terminated on day 28 after tumor cell inoculation for tumor analysis.

### Protocol for NS1 tumors

Rats inoculated with 50 000 NS1 cells were divided into two groups, one treated with only vehicle and the other with 50 mg/kg of VQ-1 starting at days–5,–3,–1, 1, 2, 4, 7, 10, and so on every third day, with inoculation of 50 000 NS1 cells done at day 0. Animals were followed until development of symptoms.

In a second experiment rats were inoculated with 5 000 NS1 cells and treated with vehicle, VQ-1 at 70 mg/kg according to the same regimen as the animals with RG2 tumors (standard treatment), or treated with VQ-1 at 70 mg/kg according to a cyclical protocol for five consecutive days preceding a 14-day washout period, after which the 5-day treatment was repeated, with treatment starting 7 days after tumor cell inoculation.

### Histological preparation

Immediately after euthanasia, brains were removed and immersed in 4% formaldehyde for a minimum of 5 days. Frozen sections were serially cut into 40μm slices focused at the location of the maximum tumor diameter in the coronal plane and stained with htx-eosin.

Other sections were incubated with a rabbit monoclonal antibody against GFAP (Antibodies online^®^), GFP (Antibodies online^®^), CD4 (Sigma-Aldrich^®^), CD8 (Antibodies online^®^) and FOXP3 (Antibodies online^®^) at a dilution of 1:200 (anti-GFAP and anti-GFP) or 1:100 (anti-CD4, anti-CD8, FOXP3) overnight and subsequently treated with a biotinylated secondary antibody and ABC reagent 30 minutes each, using a ready-to use Vectastain ABC kit (Vector Laboratories^®^, CA, USA). The antigen-antibody complex was visualized using the DAKO Liquid DAB Substrate-Chromogen System (DAKO^®^, CA, USA).

Hematoxylin-eosin stained frozen sections of the tumors were graded on a four-step semi quantitative scale describing tumor size as non-existent (0), small (1), medium (2) or large (3). A large tumor was defined as a tumor with mass effect involving more than half of the affected hemisphere, and a small tumor was defined as one identifiable under light microscope but with no mass effect. A medium sized tumor had some mass effect but occupied less than the half hemisphere. See Supplementary Figure 1 for representative example images of tumor size.

### Statistical analysis

Data analysis and visualization was done with computer, using RStudio v 1.0.136 and R v 3.3.2. Survival was described using Kaplan-Meier survival curves, and differences in survival were assessed using log-rank test. Differences in tumor size grading was tested using two-sided *t*-test assuming equal variance. Numerical measurements across groups are described as means and standard deviation. *P*-values < 0.05 were considered significant.

## CONCLUSIONS

In this study, we investigate the efficacy of VQ-1 *in vitro*, as well as orally delivered VQ-1 *in vivo* using two syngeneic rat glioblastoma models, RG2 and NS1. Although both NS1 and RG2 glioblastoma cells were affected *in vitro*, and tumor size grading was significantly lower in RG2-carrying animals receiving VQ-1 treatment, no survival advantage was apparent in rats treated with the compound compared to controls. Significant weight losses were seen in the VQ-1 treated groups. We did not see any marked difference of infiltration of CD4, CD8 or FOXP3 positive cells induced by VQ-1 treatment, indicating that VQ-1 neither increases nor decreases the host anti-tumor immune response. Further research with combined therapies and/or intratumoral VQ-1 administration using the same or other models of GBM, to minimize systemic exposure to VQ-1 while retaining tumor suppression, is recommended.

## SUPPLEMENTARY MATERIALS FIGURE


