# Supplementary material for: Evaluating vacquinol-1 in rats carrying glioblastoma models RG2 and NS1
Source: Oncotarget. 2018 Jan 3;9(9):8391–9. doi: 10.18632/oncotarget.23842 (PMC5823554; doi:10.18632/oncotarget.23842)
Supplement: Supplementary file 1 [file oncotarget-09-8391-s001.pdf]

Evaluating vacquinol-1 in rats carrying glioblastoma models RG2 and NS1

SUPPLEMENTARY MATERIALS

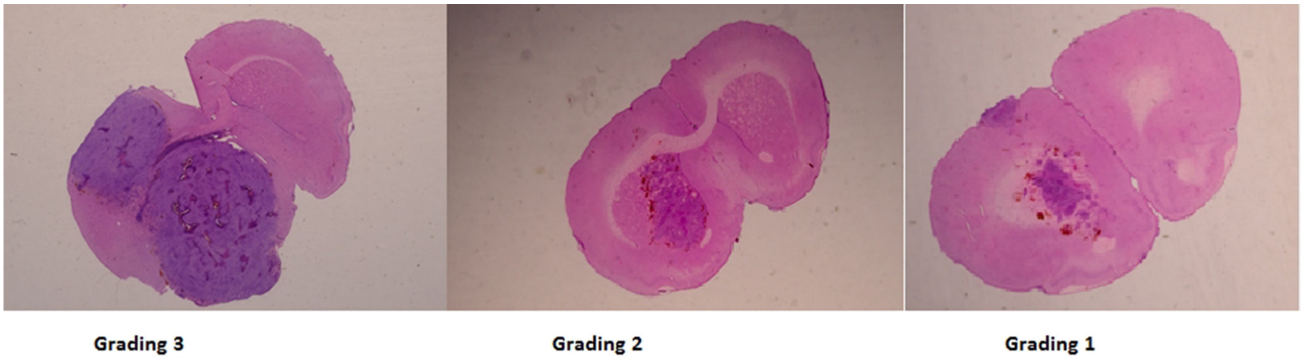

Supplementary Figure 1: Example images illustrating the semi-quantitative assessment of tumor size. From left to right, 3, 2 and 1 respectively.
